# The genome sequence of the Early Thorn, *Selenia dentaria *(Fabricius, 1775)

**DOI:** 10.12688/wellcomeopenres.20140.1

**Published:** 2023-10-20

**Authors:** Douglas Boyes, Dominic Phillips, University of Oxford and Wytham Woods Genome Acquisition Lab, Darwin Tree of Life Barcoding collective, Wellcome Sanger Institute Tree of Life programme, Wellcome Sanger Institute Scientific Operations: DNA Pipelines collective, Tree of Life Core Informatics collective, Darwin Tree of Life Consortium

**Affiliations:** 1 UK Centre for Ecology & Hydrology, Wallingford, England, UK; 2 Natural History Museum, London, England, UK

**Keywords:** Selenia dentaria, Early Thorn, genome sequence, chromosomal, Lepidoptera

## Abstract

We present a genome assembly from an individual male *Selenia dentaria* (the Early Thorn; Arthropoda; Insecta; Lepidoptera; Geometridae). The genome sequence is 1,030.8 megabases in span. Most of the assembly is scaffolded into 30 chromosomal pseudomolecules, including the Z sex chromosome. The mitochondrial genome has also been assembled and is 15.41 kilobases in length. Gene annotation of this assembly on Ensembl identified 21,390 protein coding genes.

## Species taxonomy

Eukaryota; Metazoa; Eumetazoa; Bilateria; Protostomia; Ecdysozoa; Panarthropoda; Arthropoda; Mandibulata; Pancrustacea; Hexapoda; Insecta; Dicondylia; Pterygota; Neoptera; Endopterygota; Amphiesmenoptera; Lepidoptera; Glossata; Neolepidoptera; Heteroneura; Ditrysia; Obtectomera; Geometroidea; Geometridae; Ennominae; *Selenia*; *Selenia dentaria* (Fabricius, 1775) (NCBI:txid934894).

## Background

*Selenia dentaria*, the Early Thorn, belongs to the Geometridae family of moths, with a forewing length of 14–23 mm ( Waring *et al.*, 2017). The key diagnostic feature for *S. dentaria* is its resting position, unlike other British Thorn moths, it holds its wings pressed together and upwards behind the back, resembling a butterfly ( Lewis, 2023; Waring *et al.*, 2017). The Early Thorn is variable in colour between generations and occurrence locations, but generally has 3 crosslines on the forewings with a dark staining colouration by the forewing apex ( Lewis, 2023; Waring *et al.*, 2017).

*Selenia dentaria* is a bivoltine species, with one generation from mid-February to May and a second from July to September, can be seen on the wing at dusk and readily comes to light. The larvae feed on a variety of woody broadleaved plants, including hawthorn, blackthorn, hazel and bog myrtle. The Early Thorn overwinters as a pupa spun between plant debris or leaves ( Waring *et al.*, 2017).

In the UK, *Selenia dentaria* is distributed in a variety of habitats from woodland and scrubs to urban areas and gardens; it is a common and widespread species, frequent in England, Wales, Scotland and Ireland ( Waring *et al.*, 2017). Globally, *S. dentaria* is distributed throughout northern Europe, across the Palearctic to Mongolia and the Russian far East ( GBIF Secretariat, 2023).

The larvae of *Selenia dentaria* mimic the twigs of hostplant species, e.g. hawthorn ( *Crataegus monogyna*). Previous studies have shown this is effective to avoid predation from avian predators, test trials showed that chicks who had been exposed to *S. dentaria* caterpillars and twigs of the mimicked species showed a greater latency to predate upon the caterpillar, increasing in their caution with handling twig-mimics ( Cuthill, 2019; Edmunds, 1974; Skelhorn & Ruxton, 2013; Yu *et al.*, 2022). The full genome for *S. dentaria* will give us insights into how species genetically developed these kinds of mimicry and which gene expressions or timing in gene expressions lead to their physical appearance.

This genome of *Selenia dentaria* was sequenced as part of the Darwin Tree of Life Project, a collaborative effort to sequence all named eukaryotic species in the Atlantic Archipelago of Britain and Ireland. Here we present a chromosomally complete genome sequence for *Selenia dentaria*, based on two specimens collected from Wytham Woods, Oxfordshire.

## Genome sequence report

**Figure 1.**
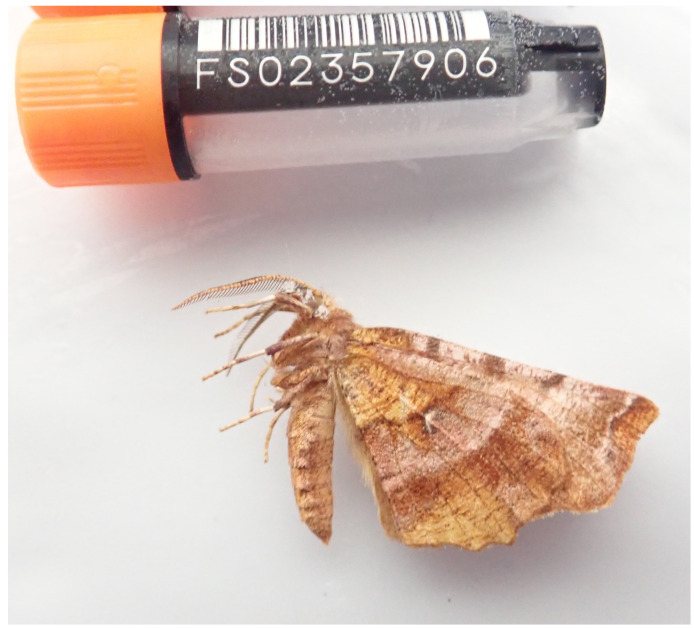
Photograph of the *Selenia dentaria* (ilSelDent1) specimen used for genome sequencing.

The genome was sequenced from one male *Selenia dentaria* ( Figure 1) collected from Wytham Woods, Oxfordshire, UK (51.77, –1.34). A total of 25-fold coverage in Pacific Biosciences single-molecule HiFi long reads and 34-fold coverage in 10X Genomics read clouds were generated. Primary assembly contigs were scaffolded with chromosome conformation Hi-C data. Manual assembly curation corrected 80 missing joins or mis-joins and removed 5 haplotypic duplications, reducing the assembly length by 0.19% and the scaffold number by 41.23%.

**Figure 2.**
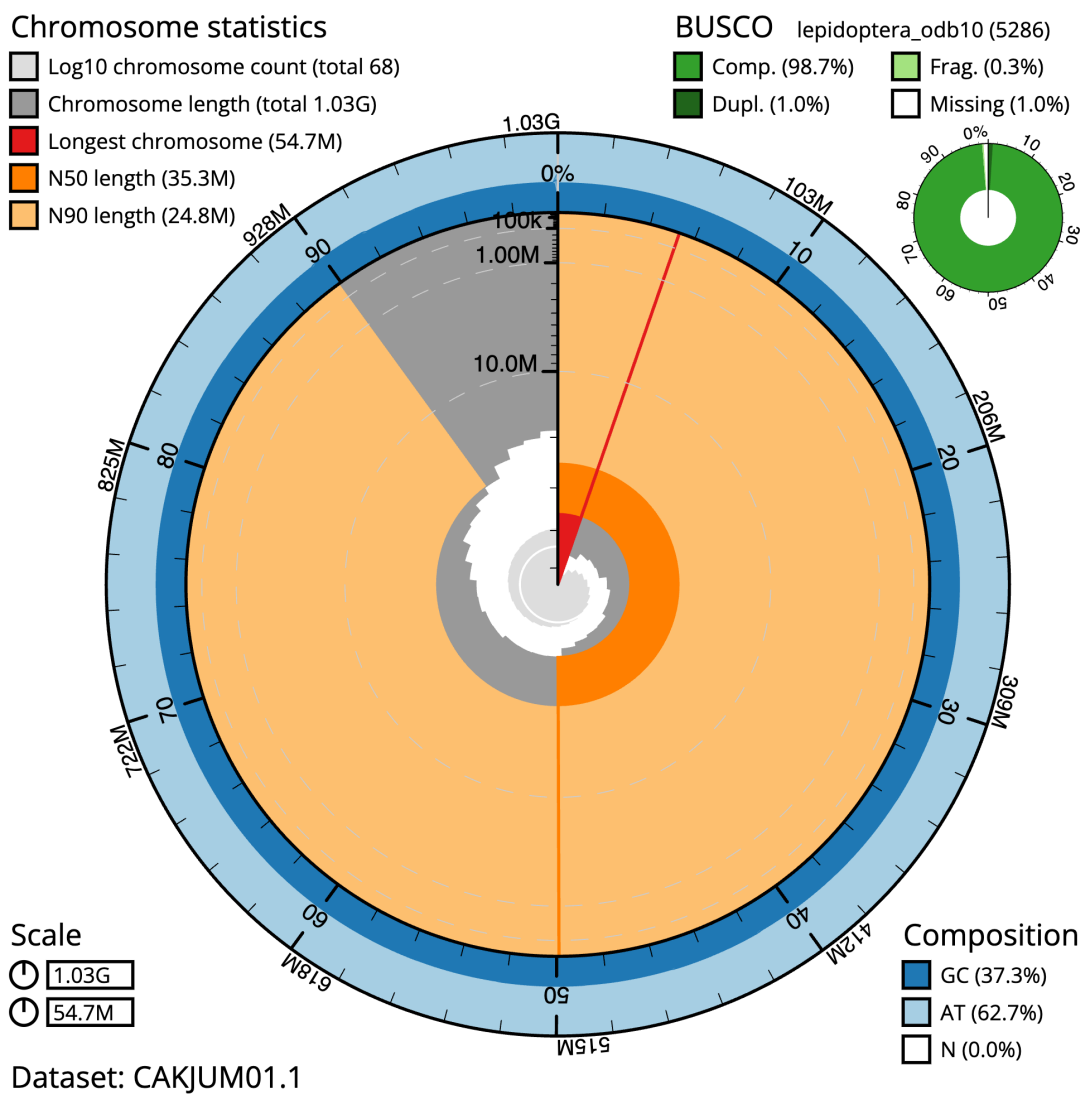
Genome assembly of *Selenia dentaria*, ilSelDent1.1: metrics. The BlobToolKit Snailplot shows N50 metrics and BUSCO gene completeness. The main plot is divided into 1,000 size-ordered bins around the circumference with each bin representing 0.1% of the 1,030,772,253 bp assembly. The distribution of scaffold lengths is shown in dark grey with the plot radius scaled to the longest scaffold present in the assembly (54,712,836 bp, shown in red). Orange and pale-orange arcs show the N50 and N90 scaffold lengths (35,319,394 and 24,754,819 bp), respectively. The pale grey spiral shows the cumulative scaffold count on a log scale with white scale lines showing successive orders of magnitude. The blue and pale-blue area around the outside of the plot shows the distribution of GC, AT and N percentages in the same bins as the inner plot. A summary of complete, fragmented, duplicated and missing BUSCO genes in the lepidoptera_odb10 set is shown in the top right. An interactive version of this figure is available at https://blobtoolkit.genomehubs.org/view/Selenia%20dentaria/dataset/CAKJUM01.1/snail.

**Figure 3.**
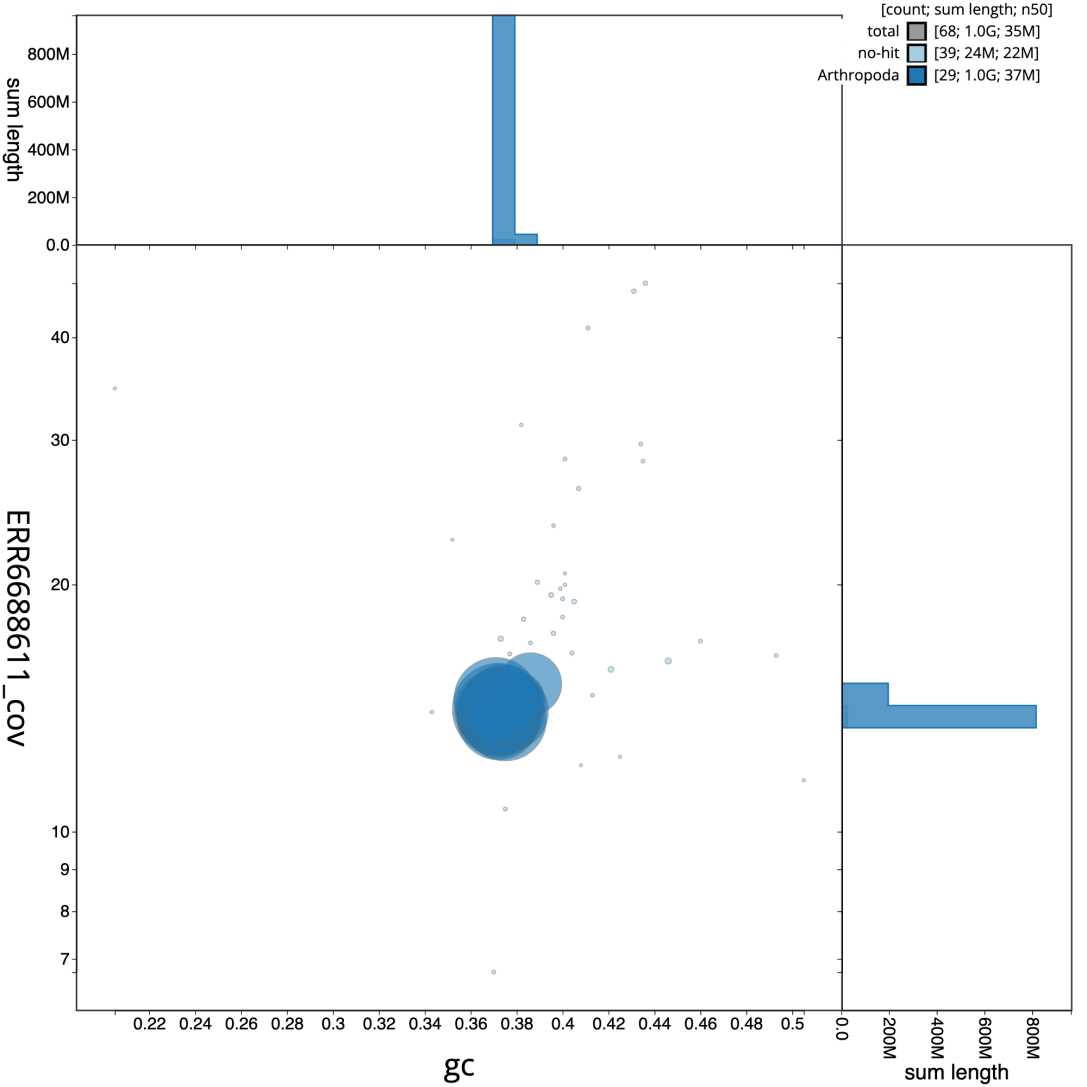
Genome assembly of *Selenia dentaria*, ilSelDent1.1: BlobToolKit GC-coverage plot. Scaffolds are coloured by phylum. Circles are sized in proportion to scaffold length. Histograms show the distribution of scaffold length sum along each axis. An interactive version of this figure is available at https://blobtoolkit.genomehubs.org/view/Selenia%20dentaria/dataset/CAKJUM01.1/blob.

**Figure 4.**
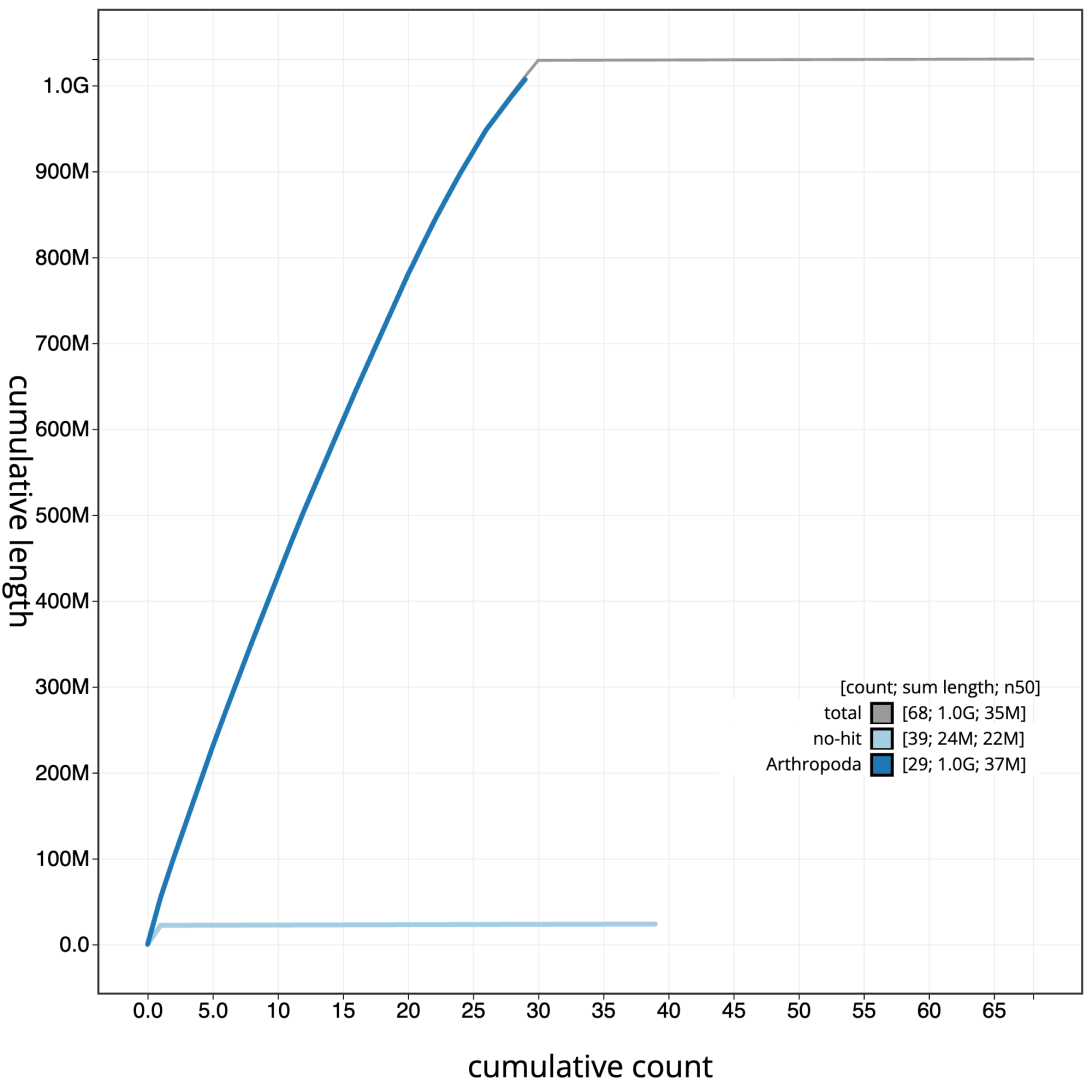
Genome assembly of *Selenia dentaria*, ilSelDent1.1: BlobToolKit cumulative sequence plot. The grey line shows cumulative length for all scaffolds. Coloured lines show cumulative lengths of scaffolds assigned to each phylum using the buscogenes taxrule. An interactive version of this figure is available at https://blobtoolkit.genomehubs.org/view/Selenia%20dentaria/dataset/CAKJUM01.1/cumulative.

**Figure 5.**
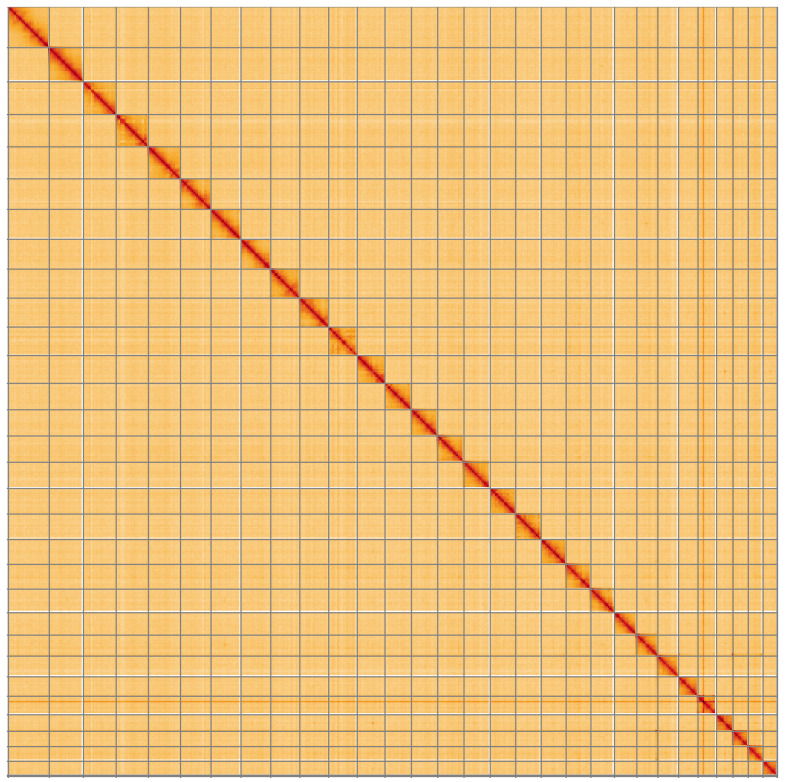
Genome assembly of *Selenia dentaria*, ilSelDent1.1: Hi-C contact map of the ilSelDent1.1 assembly, visualised using HiGlass. Chromosomes are shown in order of size from left to right and top to bottom. An interactive version of this figure may be viewed at https://genome-note-higlass.tol.sanger.ac.uk/l/?d=bMrvoXgaTr6WoTUe_xF-aQ.

The final assembly has a total length of 1,030.8 Mb in 67 sequence scaffolds with a scaffold N50 of 35.3 Mb ( Table 1). A summary of the assembly statistics is shown in Figure 2, while the distribution of assembly scaffolds on GC proportion and coverage is shown in Figure 3. The cumulative assembly plot in Figure 4 shows curves for subsets of scaffolds assigned to different phyla. Most (99.92%) of the assembly sequence was assigned to 30 chromosomal-level scaffolds, representing 29 autosomes and the Z sex chromosome. Chromosome-scale scaffolds confirmed by the Hi-C data are named in order of size ( Figure 5; Table 2). While not fully phased, the assembly deposited is of one haplotype. Contigs corresponding to the second haplotype have also been deposited. The mitochondrial genome was also assembled and can be found as a contig within the multifasta file of the genome submission.

**Table 1.** Genome data for *Selenia dentaria*, ilSelDent1.1.

Project accession data		
Assembly identifier	ilSelDent1.1	
Assembly release date	2021-11-18	
Species	*Selenia dentaria*	
Specimen	ilSelDent1	
NCBI taxonomy ID	934894	
BioProject	PRJEB46846	
BioSample ID	SAMEA7701559	
Isolate information	ilSelDent1, male: abdomen (DNA sequencing), head and thorax (Hi-C scaffolding)	
Assembly metrics *		*Benchmark*
Consensus quality (QV)	57.1	*≥ 50*
*k*-mer completeness	99.99%	*≥ 95%*
BUSCO **	C:98.7%[S:97.7%,D:1.0%], F:0.3%,M:1.0%,n:5,286	*C ≥ 95%*
Percentage of assembly mapped to chromosomes	99.92%	*≥ 95%*
Sex chromosomes	Z chromosome	*localised homologous pairs*
Organelles	Mitochondrial genome assembled	*complete single alleles*
Raw data accessions		
PacificBiosciences SEQUEL II	ERR6939253, ERR6939252	
10X Genomics Illumina	ERR6688612, ERR6688614, ERR6688611, ERR6688613, ERR7987288	
Hi-C Illumina	ERR6688610	
Genome assembly		
Assembly accession	GCA_917880725.1	
*Accession of alternate haplotype*	GCA_917880765.1	
Span (Mb)	1,030.8	
Number of contigs	160	
Contig N50 length (Mb)	20.1	
Number of scaffolds	67	
Scaffold N50 length (Mb)	35.3	
Longest scaffold (Mb)	54.7	
Genome annotation		
Number of protein-coding genes	21,390	
Number of gene transcripts	21,561	

* Assembly metric benchmarks are adapted from column VGP-2020 of “Table 1: Proposed standards and metrics for defining genome assembly quality” from ( Rhie *et al.*, 2021). ** BUSCO scores based on the lepidoptera_odb10 BUSCO set using v5.3.2. C = complete [S = single copy, D = duplicated], F = fragmented, M = missing, n = number of orthologues in comparison. A full set of BUSCO scores is available at https://blobtoolkit.genomehubs.org/view/Selenia%20dentaria/dataset/CAKJUM01.1/busco.

**Table 2.** Chromosomal pseudomolecules in the genome assembly of *Selenia dentaria*, ilSelDent1.

INSDC accession	Chromosome	Length (Mb)	GC%
OU862875.1	1	54.71	37.2
OU862877.1	2	43.87	37.5
OU862878.1	3	43.21	37.2
OU862879.1	4	42.88	37.3
OU862880.1	5	40.94	37.2
OU862881.1	6	40.05	37.2
OU862882.1	7	39.94	37.4
OU862883.1	8	38.89	37.1
OU862884.1	9	38.69	37
OU862885.1	10	38.18	37.7
OU862886.1	11	37.25	37.1
OU862887.1	12	35.32	37.4
OU862888.1	13	35.25	37
OU862889.1	14	35.15	37.2
OU862890.1	15	35.1	37.2
OU862891.1	16	34.23	37.2
OU862892.1	17	34.13	37.2
OU862893.1	18	33.33	37.2
OU862894.1	19	33.16	37.3
OU862895.1	20	31.6	37.3
OU862896.1	21	29.96	37.4
OU862897.1	22	28.13	37.7
OU862898.1	23	27.65	37.3
OU862899.1	24	26.13	37.3
OU862900.1	25	24.75	38.6
OU862901.1	26	22.23	37.1
OU862902.1	27	20.37	37.6
OU862903.1	28	19.76	38
OU862904.1	29	18.63	37.8
OU862876.1	Z	45.76	37.1
OU862905.1	MT	0.02	20.8

The estimated Quality Value (QV) of the final assembly is 57.1 with *k*-mer completeness of 99.99%, and the assembly has a BUSCO v5.3.2 completeness of 98.7% (single = 97.7%, duplicated = 1.0%), using the lepidoptera_odb10 reference set ( *n* = 5,286).

Metadata for specimens, spectral estimates, sequencing runs, contaminants and pre-curation assembly statistics can be found at https://links.tol.sanger.ac.uk/species/934894.

## Genome annotation report

The *Selenia dentaria* genome assembly (GCA_917880725.1) was annotated using the Ensembl rapid annotation pipeline ( Table 1; https://rapid.ensembl.org/Selenia_dentaria_GCA_917880725.1/Info/Index). The resulting annotation includes 21,561 transcribed mRNAs from 21,390 protein-coding genes.

## Methods

### Sample acquisition and nucleic acid extraction

A male *Selenia dentaria* (specimen ID Ox000698, ToLID ilSelDent1) was collected from Wytham Woods, Oxfordshire (biological vice-county Berkshire), UK (latitude 51.77, longitude –1.34) on 2020-07-20 using a light trap. The specimen was collected and identified by Douglas Boyes (University of Oxford) and preserved on dry ice.

DNA was extracted at the Tree of Life laboratory, Wellcome Sanger Institute (WSI). The ilSelDent1 sample was weighed and dissected on dry ice with head and thorax tissue set aside for Hi-C sequencing. Abdomen tissue was disrupted using a Nippi Powermasher fitted with a BioMasher pestle. High molecular weight (HMW) DNA was extracted using the Qiagen MagAttract HMW DNA extraction kit. Low molecular weight DNA was removed from a 20 ng aliquot of extracted DNA using the 0.8X AMpure XP purification kit prior to 10X Chromium sequencing; a minimum of 50 ng DNA was submitted for 10X sequencing. HMW DNA was sheared into an average fragment size of 12–20 kb in a Megaruptor 3 system with speed setting 30. Sheared DNA was purified by solid-phase reversible immobilisation using AMPure PB beads with a 1.8X ratio of beads to sample to remove the shorter fragments and concentrate the DNA sample. The concentration of the sheared and purified DNA was assessed using a Nanodrop spectrophotometer and Qubit Fluorometer and Qubit dsDNA High Sensitivity Assay kit. Fragment size distribution was evaluated by running the sample on the FemtoPulse system.

### Sequencing

Pacific Biosciences HiFi circular consensus and 10X Genomics read cloud DNA sequencing libraries were constructed according to the manufacturers’ instructions. DNA sequencing was performed by the Scientific Operations core at the WSI on Pacific Biosciences SEQUEL II (HiFi) and Illumina HiSeq 4000 and Illumina NovaSeq 6000 (10X) instruments. Hi-C data were also generated from head and thorax tissue of ilSelDent1 using the Arima2 kit and sequenced on the Illumina NovaSeq 6000 instrument.

### Genome assembly, curation and evaluation

Assembly was carried out with Hifiasm ( Cheng *et al.*, 2021) and haplotypic duplication was identified and removed with purge_dups ( Guan *et al.*, 2020). One round of polishing was performed by aligning 10X Genomics read data to the assembly with Long Ranger ALIGN, calling variants with FreeBayes ( Garrison & Marth, 2012). The assembly was then scaffolded with Hi-C data ( Rao *et al.*, 2014) using SALSA2 ( Ghurye *et al.*, 2019). The assembly was checked for contamination and corrected as described previously ( Howe *et al.*, 2021). Manual curation was performed using HiGlass ( Kerpedjiev *et al.*, 2018) and Pretext ( Harry, 2022). The mitochondrial genome was assembled using MitoHiFi ( Uliano-Silva *et al.*, 2023), which runs MitoFinder ( Allio *et al.*, 2020) or MITOS ( Bernt *et al.*, 2013) and uses these annotations to select the final mitochondrial contig and to ensure the general quality of the sequence.

A Hi-C map for the final assembly was produced using bwa-mem2 ( Vasimuddin *et al.*, 2019) in the Cooler file format ( Abdennur & Mirny, 2020). To assess the assembly metrics, the *k*-mer completeness and QV consensus quality values were calculated in Merqury ( Rhie *et al.*, 2020). This work was done using Nextflow ( Di Tommaso *et al.*, 2017) DSL2 pipelines “sanger-tol/readmapping” ( Surana *et al.*, 2023a) and “sanger-tol/genomenote” ( Surana *et al.*, 2023b). The genome was analysed within the BlobToolKit environment ( Challis *et al.*, 2020) and BUSCO scores ( Manni *et al.*, 2021; Simão *et al.*, 2015) were calculated.

Table 3 contains a list of relevant software tool versions and sources.

**Table 3.** Software tools: versions and sources.

Software tool	Version	Source
BlobToolKit	4.0.7	https://github.com/blobtoolkit/blobtoolkit
BUSCO	5.3.2	https://gitlab.com/ezlab/busco
FreeBayes	1.3.1-17- gaa2ace8	https://github.com/freebayes/freebayes
Hifiasm	0.15.3	https://github.com/chhylp123/hifiasm
HiGlass	1.11.6	https://github.com/higlass/higlass
Long Ranger ALIGN	2.2.2	https://support.10xgenomics.com/genome-exome/ software/pipelines/latest/advanced/other-pipelines
Merqury	MerquryFK	https://github.com/thegenemyers/MERQURY.FK
MitoHiFi	2	https://github.com/marcelauliano/MitoHiFi
PretextView	0.2	https://github.com/wtsi-hpag/PretextView
purge_dups	1.2.3	https://github.com/dfguan/purge_dups
SALSA	2.2	https://github.com/salsa-rs/salsa
sanger-tol/ genomenote	v1.0	https://github.com/sanger-tol/genomenote
sanger-tol/ readmapping	1.1.0	https://github.com/sanger-tol/readmapping/tree/1.1.0

### Genome annotation

The BRAKER2 pipeline ( Brůna *et al.*, 2021) was used in the default protein mode to generate annotation for the *Selenia dentaria* assembly (GCA_917880725.1) in Ensembl Rapid Release.

### Wellcome Sanger Institute – Legal and Governance

The materials that have contributed to this genome note have been supplied by a Darwin Tree of Life Partner. The submission of materials by a Darwin Tree of Life Partner is subject to the **‘Darwin Tree of Life Project Sampling Code of Practice’**, which can be found in full on the Darwin Tree of Life website here. By agreeing with and signing up to the Sampling Code of Practice, the Darwin Tree of Life Partner agrees they will meet the legal and ethical requirements and standards set out within this document in respect of all samples acquired for, and supplied to, the Darwin Tree of Life Project.

Further, the Wellcome Sanger Institute employs a process whereby due diligence is carried out proportionate to the nature of the materials themselves, and the circumstances under which they have been/are to be collected and provided for use. The purpose of this is to address and mitigate any potential legal and/or ethical implications of receipt and use of the materials as part of the research project, and to ensure that in doing so we align with best practice wherever possible. The overarching areas of consideration are:

•   Ethical review of provenance and sourcing of the material

•   Legality of collection, transfer and use (national and international)

Each transfer of samples is further undertaken according to a Research Collaboration Agreement or Material Transfer Agreement entered into by the Darwin Tree of Life Partner, Genome Research Limited (operating as the Wellcome Sanger Institute), and in some circumstances other Darwin Tree of Life collaborators.

## Data Availability

European Nucleotide Archive: *Selenia dentaria* (early thorn). Accession number PRJEB46846; https://identifiers.org/ena.embl/PRJEB46846. ( Wellcome Sanger Institute, 2021) The genome sequence is released openly for reuse. The *Selenia dentaria* genome sequencing initiative is part of the Darwin Tree of Life (DToL) project. All raw sequence data and the assembly have been deposited in INSDC databases. Raw data and assembly accession identifiers are reported in Table 1.

## Author information

Members of the University of Oxford and Wytham Woods Genome Acquisition Lab are listed here: https://doi.org/10.5281/zenodo.4789928.

Members of the Darwin Tree of Life Barcoding collective are listed here: https://doi.org/10.5281/zenodo.4893703.

Members of the Wellcome Sanger Institute Tree of Life programme are listed here: https://doi.org/10.5281/zenodo.4783585.

Members of Wellcome Sanger Institute Scientific Operations: DNA Pipelines collective are listed here: https://doi.org/10.5281/zenodo.4790455.

Members of the Tree of Life Core Informatics collective are listed here: https://doi.org/10.5281/zenodo.5013541.

Members of the Darwin Tree of Life Consortium are listed here: https://doi.org/10.5281/zenodo.4783558.
