# Supplementary material for: The genome sequence of the Early Thorn, Selenia dentaria (Fabricius, 1775)
Source: Wellcome Open Res. 2023 Oct 20;8:485. [Version 1] doi: 10.12688/wellcomeopenres.20140.1 (PMC11066531; doi:10.12688/wellcomeopenres.20140.1)
Supplement: Peer review history

# Reviewer report: Reviewer response for version 1

**DOI:** 10.21956/wellcomeopenres.22302.r78098

**Published:** 2024-05-02

**Authors:** Kuppusamy Sivasankaran

**Affiliations:** 1 Entomology Research Institute, Loyola College, Chennai, Tamil Nadu, India

I appreciate the authors for assembling the genome sequence of Early Thorn, *Selenia dentaria *(Fabricius, 1775). Using 67 scaffolds, the authors sequenced approximately 1030.8 mega bases of the genome assembly. They have used standard methods for DNA isolation and followed appropriate software for the sequence assembly and annotations.  

 The minor clarification is given below.

 In the background of the article authors have given the genus name *Selenia dentaria* full name and short name continuously. First time the genus name can be given in full name then subsequently can be given in short form like *S. dentaria*.

 In the background third line “can be seen on the wing at dusk and readily comes to light”. This message is not appropriate here and confusing. The sentence can be deleted or elaborate meaningfully.

 In the page no 6 para second the sentence can be modified as DNA was extracted at the Darwin Tree of Life laboratory and Wellcome Sanger Institute (WSI).

 Overall, I confirm that the article is accepted for scientific standard

Are sufficient details of methods and materials provided to allow replication by others?

Yes

Is the rationale for creating the dataset(s) clearly described?

Yes

Are the datasets clearly presented in a useable and accessible format?

Yes

Are the protocols appropriate and is the work technically sound?

Yes

Reviewer Expertise:

Phylogenetic analysis of Noctuoidea moths using mitogenome sequence

I confirm that I have read this submission and believe that I have an appropriate level of expertise to confirm that it is of an acceptable scientific standard.

# Reviewer report: Reviewer response for version 1

**DOI:** 10.21956/wellcomeopenres.22302.r73463

**Published:** 2024-02-27

**Authors:** Jullien M Flynn

**Affiliations:** 1 Whitehead Institute for Biomedical Research, Cambridge, MA, USA

In this Data Note, the authors present a genome assembly for *Selenia dentaria*, as part of the Darwin Tree of Life Project. The genome assembly is of high quality and the figures shown are informative. The assembly is publicly available. 

 I only have a couple of minor suggestions for clarity:

 1) It is mentioned that the assembly is based on two specimens, but DNA extraction and sequencing is described to be only from a single male. Please clarify. 

 2) The last part of this sentence is unclear: " *Selenia dentaria* is a bivoltine species, with one generation from mid-February to May and a second from July to September, can be seen on the wing at dusk and readily comes to light." It is not clear **what** can be seen on the wing at dusk. Please separate this last idea into a new sentence or show how it is clearly connected.

Are sufficient details of methods and materials provided to allow replication by others?

Yes

Is the rationale for creating the dataset(s) clearly described?

Yes

Are the datasets clearly presented in a useable and accessible format?

Yes

Are the protocols appropriate and is the work technically sound?

Yes

Reviewer Expertise:

Evolutionary genomics

I confirm that I have read this submission and believe that I have an appropriate level of expertise to confirm that it is of an acceptable scientific standard.
